# The differential needs and expectations from general practitioners in oncology between high-income countries and low- and-middle-income countries: results from a survey of Canadian and Nepali oncologists

**DOI:** 10.3332/ecancer.2024.1673

**Published:** 2024-02-22

**Authors:** Bishal Gyawali, Bishesh Sharma Poudyal, Laura M Carson, Colleen Savage, Ramila Shilpakar, Scott Berry

**Affiliations:** 1 Department of Oncology, Queen’s University, Kingston, ON K7L 3N6, Canada; 2 Division of Cancer Care and Epidemiology, Queen’s Cancer Research Institute, Kingston, ON K7L 3N6, Canada; 3 Department of Public Health Sciences, Queen’s University, Kingston, ON K7L 3N6, Canada; 4 Department of Hematology, Civil Service Hospital, Kathmandu 44600, Nepal; 5 Department of Clinical Oncology, National Academy of Medical Sciences, Bir Hospital, Kathmandu 44600, Nepal

**Keywords:** oncology, global oncology, general practitioners, general practitioners in oncology, Nepal, Canada

## Abstract

**Background:**

To address the shortage of oncologists in the wake of the rapidly increasing global cancer burden, general practitioners of oncology (GPOs) have been added to cancer care teams worldwide. GPOs are family physicians with additional training in oncology and their roles differ by both country and region. In this study, we aimed to learn about the roles and expectations of GPOs from the perspective of oncologists in Canada and Nepal.

**Methods:**

A survey was designed and administered to Canadian and Nepali Oncologists between February and November 2022 using Research Electronic Data Capture, a secure web-based software platform hosted at Queen’s University in Kingston, Ontario, Canada. Participants were recruited through personal networks/social media in Nepal and the survey was distributed through an email list provided by the Canadian Association of Medical Oncologists.

**Results:**

The survey received 48 responses from Canadian and 7 responses from Nepali oncologists. Canadian respondents indicated that in terms of educational content delivery, clinics with oncologists followed by didactic lectures by oncologists were thought to be the most effective, followed by a small group learning and online education. Nepali oncologists also indicated didactic lectures by oncologists and small group learning would be the most effective teaching techniques, followed by online education and clinics with oncologists. Critical knowledge domains and skills most relevant for GPO training identified by Canadian respondents were managing pain and other common symptoms of cancers, as well as treatment of common side effects, followed by goals of care discussion, post-treatment surveillance for recurrence, and the management of long-term complications from treatment. Respondents from Nepal, however, suggested an approach to diagnosis to patient with increased risk of cancer, and cancer staging were the most critical knowledge domains and skills. The majority of oncologists in both countries thought a training program of 6–12 months was optimal.

**Conclusion:**

We found many similarities in oncologist’s opinions of GPOs between the two countries, however, there were also some notable differences such as the need to provide cancer screening services in Nepal. This highlights the need to tailor GPO training programs based on local context.

## Introduction

Many countries face a significant shortage of cancer care providers, including medical oncologists, despite a simultaneous rising burden of cancer [1, 2]. Since training a medical student to become a medical oncologist takes substantial time, money, and resources, increasing the number of medical oncologists will not be a sufficient solution to address this shortage for many health systems in the world. To mitigate this challenge, countries such as Canada have started to train general practitioners (GPs) in cancer care, referred to as general practitioners of oncology (GPOs), as part of a task-shifting and task-sharing model of cancer care. GPOs are family physicians that have received specialised training to provide specific cancer care services in collaboration with cancer specialists, including supervision of systemic therapy, and pain and symptom management.

In Canada, the GPO role was introduced over 40 years ago and they have been integrated into national health systems as a strategy to expand and strengthen a country’s cadre of cancer care providers and have been found to offer significant value [3]. There is also a national association of GPOs in Canada called the Canadian Association of General Practitioners in Oncology. In Nepal, there is no formal, nationally integrated GPO training program yet [4]. A lack of trained cancer care providers has been identified as one of the most significant challenges in Nepal to providing high-quality cancer care, highlighting an urgent need to strengthen their workforce [5]. Currently, only one institution in Nepal offers a medical oncology training fellowship. Furthermore, there are no incentives for medical oncologists to practice outside of urban areas. This gap can be partly addressed by training GPOs who can also improve geographic accessibility to cancer care. For example, the centralised nature of cancer care in Nepal limits the ability of patients living in rural regions to access timely care [5]. Many patients are forced to travel long distances to access screening, diagnosis, treatment, or palliative services which imposes significant time and financial toxicity on to patients and their caregivers. The integration of GPOs in Nepal may be a prospective approach to increase the geographic availability of cancer care.

We took a systematic approach to informing the development of a comprehensive curriculum to train GPOs in Nepal. First, we conducted a scoping review to identify existing curricula available globally for training GPOs, and found a dearth of systematic training curricula [3]. Second, we did a survey of Nepali GPs and identified a strong interest, need, and support for a national GPO training program in Nepal [6]. Third, we surveyed Canadian GPOs to understand from their training and work experience regarding the effective training modalities, and subsequent practice patterns [7]. The purpose of this current study, which is the final step in this process before drafting the curriculum, was to conduct a survey among oncologists in Nepal and Canada to draw comparisons about the experiences, perceptions, and preferences of oncologists in each country regarding GPO training and collaboration. Understanding these differences or similarities will guide the design and implementation of a novel GPO training program in Nepal, providing critical insights for future projects in other countries and contexts. Our findings will also help clarify the role and expectations of GPOs in current settings.

## Methods

A survey was designed and distributed to Canadian and Nepali oncologists using Research Electronic Data Capture (REDCap), a secure web-based software platform hosted at Queen’s University in Kingston, Ontario, Canada. The survey was granted ethics approval by the Queen’s University Health Sciences and Affiliated Teaching Hospitals Research Ethics Board.

The survey consisted of the following sections: respondent demographics; current scope of oncology practice; existing and future GPO services; oncology teams at their institution; knowledge and skills deemed important for GPOs; oncology-related services available at their institution; and open feedback on GPOs. A copy of the survey can be found in the Supplemental Appendix.

The survey was opened in February 2022 and remained active until November 2022. Participants were recruited in Nepal through personal email communication and social media networks such as Twitter. Canadian participants were recruited through the Canadian Association of Medical Oncologists (CAMO) network mailing list that included two follow-up reminders. Survey respondents were oncologists who were currently working in Canada or Nepal. The participants were aware that the purpose of the survey was to inform the development of a GPO training curriculum for Nepal.

Responses were recorded in REDCap and analysed in Microsoft Excel. Descriptive statistics were reported in this paper.

Patients and the public were not involved in the design and conduct of this study.

## Results

### Demographics

Of the 208 medical oncologists in Canada who received our invitation to participate in the survey through the CAMO mailing list, 48 responded for an estimated response rate of 23%. In Nepal, there are approximately 27 medical oncologists and we received responses from 7 for an estimated respond rate of 26%. Table 1 shows the demographic information of the respondents. In Canada, 54% of respondents were female and 52% were over age 45. In Nepal, 57% of respondents were female and all were between 35 and 45 years old. In our Canadian sample, two-thirds of respondents had been practicing as an oncologist for less than 20 years, and a third had been practicing for more than 20 years. In contrast, in Nepal, most oncologists (86%) had been practicing for less than 10 years and none had been practicing for more than 20 years.

Almost all Canadian oncologists who responded (96%) practiced in a large population centre with over 100,000 inhabitants. Similarly, in Nepal, all respondents served in a Metropolitan area. While nearly all Canadian oncologists (98%) were affiliated with a medical college, only 71% of those in Nepal were affiliated. All the Nepali respondents worked in a public/government institution, as did most (94%) of the Canadian oncologists. All the respondents in both countries participated in regular multi-disciplinary case conferences.

### Oncology practice

Data was collected on the current scope of clinical practice of respondents. In Canada, 85% of oncologists indicated that their current practice was ‘mostly out-patient’. In Nepal, the majority of oncologists (57%) had an equal practice burden between in-patients and out-patients. Most Canadian respondents were responsible for between 1 and 5 inpatients (77% of respondents) and 11–15 outpatients (44% of respondents) on an average day in Canada, while oncologists in Nepal typically cared for 30 patients during a typical out-patient clinic day. The commonest cancers to be presented at their clinics were gastrointestinal (31%), lung (27%), and breast (19%) for Canadian respondents and gastrointestinal (43%), lung (43%), and haematological (14%) for Nepali respondents. No one in either country indicated cervical, prostate, head/neck or skin as the most common tumour type presenting at their clinic.

### GPO services

In Canada, 75% of oncologists reported having a GPO working with them. In Nepal, when asked if there was a need for a GPO training program, two-thirds of oncologists indicated there was a need for a program in Nepal and 50% responded that they would like to have a GPO working with them.

**Figure 1.**
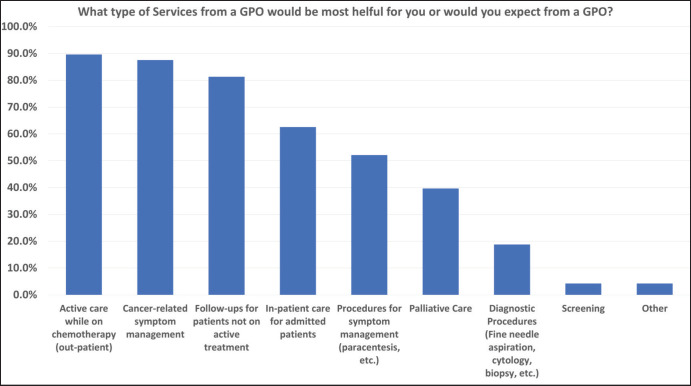
Canada- GPO cancer services most helpful.

**Figure 2.**
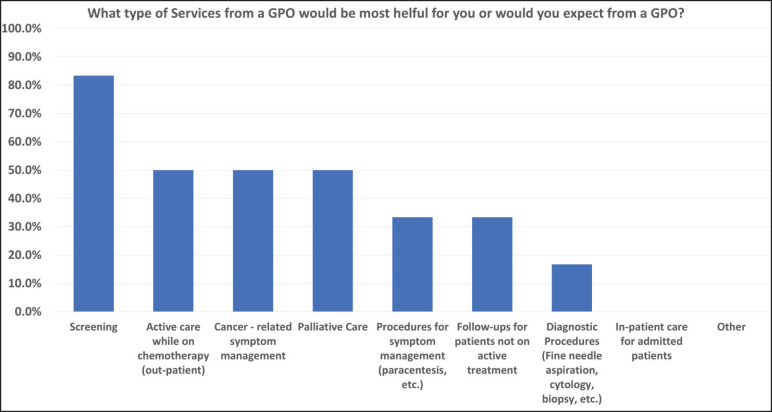
Nepal- GPO cancer services most helpful.

In terms of what services would be most helpful from a GPO, Canadian oncologists indicated actively caring for out-patients while on chemotherapy, cancer-related symptom management, and following up patients not on active treatment were the most valuable. The three least useful services provided were screening, diagnostic procedures, and palliative care (Figure 1). In contrast for Nepal, screening, actively caring for out-patients while on chemotherapy,

cancer-related symptom management and palliative care were thought to be the most helpful services GPOs could provide, while diagnostic procedures, performing procedures for symptom management, and following up patients not on active treatment were the least useful (Figure 2).

### Oncology teams

In terms of the composition of their professional teams, 98% of Canadian oncologists reported they had nurses, pharmacists, and medical oncologists on their teams. In Nepal, all the oncologists had nurses on their team and 83% also had pharmacists and surgical oncologists. Least frequently listed as team members in Canada were occupational therapists (31%), psychologists (40%), and haematologists (60%). In Nepal, social workers (33%) were the least common team members, and only half reported working with occupational therapists, psychologists, nurse practitioners or radiation oncologists.

### GPO skills

Canadian and Nepal oncologists both agreed that the most important skills for a GPO were paracentesis, thoracocentesis, and bone marrow biopsies. Fine needle aspiration cytology and peripherally inserted central cathether (PICC) line insertion were the least important in both countries.

With regards to clinical and communication skills, the most important ones in Canada were managing pain and other cancer symptoms, managing common treatment side effects, goals of care discussion and post-treatment surveillance for recurrence. The least important skill was approaching a patient with increased risk of cancer. In contrast in Nepal, approach to diagnosis, approach to patient with increased risk of cancer and approach to staging cancer were identified as the most important skills, while treating common cancers was the least important.

### GPO knowledge

For the knowledge domains critical in a GPO’s practice, the most important ones in Canada were treatment of side effects of cancer treatment, symptom management protocols and side effects of cancer treatment, oncology emergencies. The least important were the epidemiology of common cancers, screening for common cancers, knowledge of hereditary cancers and when to refer for genetic assessment. In Nepal, respondents felt it was most important for GPOs to know the epidemiology of common cancers and how to screen for common cancers, while knowing treatment protocols for common cancers and survivorship care were the least important knowledge domains.

### Access of services

**Figure 3.**
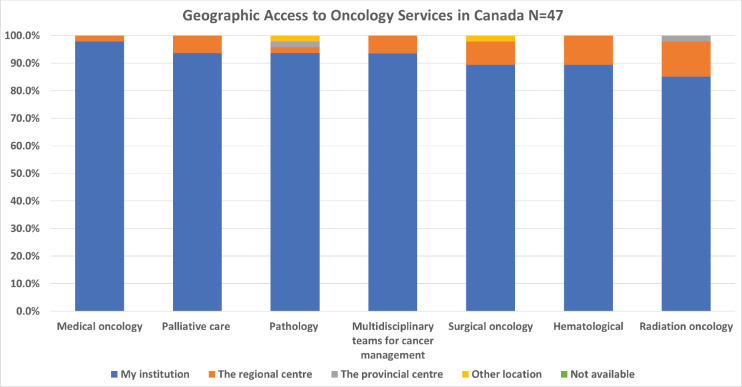
Canadian oncologist’s geographic access to services.

**Figure 4.**
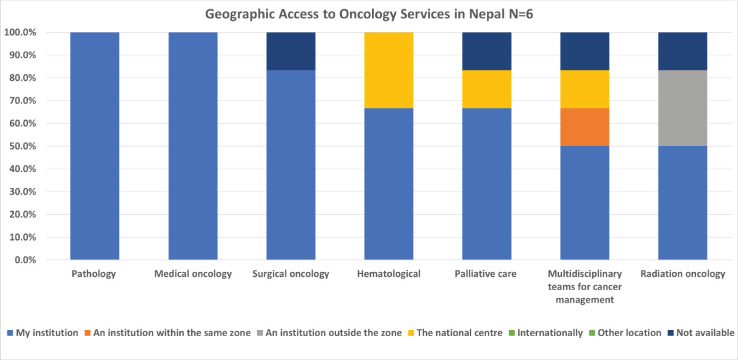
Nepalese oncologist’s geographic access to services.

Survey participants also reported on geographic access to oncology services. The services most available within the institution of the respondents in Canada were medical oncology, palliative care, and pathology, while the least readily available were radiation oncology, haematology, and surgical oncology (Figure 3). In Nepal however, the most available services were pathology, medical oncology and surgical oncology, while the least readily available were radiation oncology, multidisciplinary teams and palliative care (Figure 4).

### Open feedback

Among the Nepali respondents, concerns for the division of responsibilities between GPOs and oncologists were apparent. Specifically, malpractice related to the incorrect prescription of treatment regimens and medications was noted as a potential challenge for a GPO training program, with concerns that GPOs may extend their clinical practice beyond their training. Among the Canadian respondents, although one respondent warned against having GPOs responsible for in and out-patient care simultaneously and recommended having separate positions for these care modalities, several respondents emphasised the value and autonomy of GPOs in their practice. One participant claimed they are ‘invaluable members of [the] oncology health care team’, and another stated that in less accessible communities GPOs ‘may know the patient best and be viewed as the “centre” of the patient’s care team’.

## Discussion

Our survey of Canadian and Nepali oncologists highlighted critical differences in oncologists’ experiences and perspectives regarding the importance of various GPOs services. Despite some similarities in the expected roles of GPOs, this survey has highlighted some key differences between the expectations from GPOs in Canada versus Nepal, which underscores the need to tailor the GPO training program and post-training work opportunities based on the local need and context. For example, screening and staging of cancers were deemed as major GPO responsibilities in Nepal, but not in Canada.

There was a substantial disparity in the perception of screening as a GPO responsibility. Among Nepali respondents, 83% indicated that screening was a helpful or expected service which is in stark contrast to only 4% of the Canadian respondents who expected screening services from GPOs. This emphasises the relevance of examining health system differences and highlights the need to adapt training programs in a local context. While screening would not need to be a GPO priority in Canada as family physicians already fulfil this role [8], in Nepal, GPOs could dedicate substantial time and effort in assessing patients for the presence of cancer as there is an unmet need for routine screening [9]. In contrast to screening, two GPO services highly ranked among both groups where cancer-related symptom management and active care while on chemotherapy which underscores their common value in GPO services.

Differences were also found regarding perceptions of communication skills deemed important for a GPO. Approach to patients with increased risk of cancer was indicated as not important by 23% of Canadian respondents, but none of Nepali respondents. This difference may reflect variations in patient population and health systems as mentioned before since there is more of a need for routine screening in Nepal [9, 10]. Also, cultural customs and norms may influence communication styles [11] and this finding should also be understood in the context of the teams with which respondents work. When asked about multidisciplinary collaboration, 83% of Canadian respondents reported working with social workers, in comparison to only 33% of Nepali respondents. The general absence of social workers in Nepali practice may cause a shift in the responsibilities for communication and emotional support onto physicians, heightening the relevance of GPO supportive communication skills [12]. Team composition impacts GPO roles and responsibilities. The relevance of multidisciplinary engagement was apparent as both Canadian and Nepali oncologists agreed most strongly that ‘working with other physicians and healthcare providers to provide multidisciplinary care’ was critical to the GPO discipline. This suggests that collaboration is a critical skill in the oncology profession with universal importance.

Some differences in what should be included in a GPO’s knowledge base were evident. In Nepal, the important ones were understanding the epidemiology of common cancers and screening for cancers. In Canada, knowing how to handle the side effects of radiation and systemic therapy toxicities and the management protocols for the symptoms and side effects of treatment were most valuable. For Nepal, the emphasis on screening and epidemiology aligns with previous reports that found a lack of awareness of cancer prognosis can lead to delayed presentation and diagnosis, which in part explained the high proportion of advanced stage cancer cases [13].

Clinical skills most valued by oncologists in Nepal also differed from those in Canada. In Canada, managing pain and other symptoms of cancer were most relevant, and managing treatment side effects was important for both countries. In Nepal, approaches to diagnosis and staging of cancer were reported as valuable skills and this may again be due to the high rates of advanced-stage cancer at presentation [14].

Notably, preferences for GPO training program design differed slightly. While the majority of respondents in both countries indicated a preference for a training program of 6–12 months, nearly 30% of Canadian respondents opted for a 3–6 months program, whereas none of the Nepal oncologists preferred this shorter program. A longer training timeframe may need to be considered in designing future programs for the Nepali setting.

The availability of cancer-related services varied between countries, but some similarities existed. For both countries, radiation oncology was one of the three least available services, and medical oncology and pathology were the most readily available at their own institution. One clear distinction, however, was that no Canadian respondents selected ‘not available’ for any of the listed services listed. In Nepal, one respondent indicated that radiation oncology, multidisciplinary teams for cancer management, palliative care, and surgical oncology were all not available. This health inequity must be considered when implementing training programs. An assessment of where students can train with adequate equipment and accessible services needs to be assimilated into the GPO educational plan. In addition, once again, the integration of multidisciplinary teams appears to be less common in Nepal, highlighting this as a potential area for further development.

The open feedback section of the survey offered new insights on the perspectives and experiences of GPO training in the context of each country. Nepali respondents expressed concern surrounding GPO’s scope of practice, overlap with med oncologists, and liabilities when practicing outside the scope of practice. These issues will be critical to address as plans are made to launch a national GPO training program in Nepal. Ensuring program uptake will be contingent on the support and encouragement of oncologists, GPs, government stakeholders, and other oncology healthcare providers. Opportunities for open dialogue to discuss concerns, questions, or reservations will be necessary to facilitate clear communication and address issues noted in our surveys. Furthermore, continued supervision of the program and practice standards of the graduates maybe needed for a certain number of years to ensure that quality of cancer care has remained optimal. This would generate greater buy in from the regulators in other low-and-middle-income countries as well. The tone of Canadian respondents, on the other hand, was mostly positive, and expressed acknowledgment of the significant role that GPOs play in Canadian cancer care system. Indeed, previous studies have demonstrated the invaluable role of GPOs in Canadian cancer care system [15, 16]. This sets a precedent that GPO training can successfully be integrated into health systems and with adequate planning, the challenges expressed by Nepali oncologists maybe addressed and surmounted.

One clear limitation of this study is that the Nepali sample size is quite small (*N* = 7). However, this is a natural reflection of the relatively small cancer care workforce in the country. Further, a lack of rural representation from both Canadian and Nepali respondents reduces generalisability of the findings. Again, however, there are no cancer centres in rural Nepal and thus, all medical oncologists work in urban settings. The age and gender distribution of the respondents in our survey is consistent with the 2020 national cohort in which 46% of Canadian medical oncologists were female, and 40% were over the age of 50 [17]. In Nepal, 100% of respondents fell between the ages of 35–44, whereas the Canadian group had over 50% of participants over the age of 44. Similarly, over 70% of Nepali respondents had been practicing as an oncologist for less than 5 years, compared to only 25% in Canada. This finding is expected given that medical oncology is a relatively new discipline in Nepal. Older oncologists may have had more time to work with GPOs and see challenges resolved that the younger group in Nepal had not yet experienced and this could have artificially created a contrast in the support for GPOs between the countries. Lastly, a Canadian medical oncology network was utilised to distribute the survey, whereas Nepali respondents were recruited through personal networks and social media, and this differences in recruitment methods could impact our results.

## Conclusion

While similarities exist in some aspects of the survey, the topics and skills most worthwhile for GPOs demonstrated some central differences in the perceptions and experiences of the GPO role among Canadian and Nepali oncologists. These differences underscore the need to contextualise GPO training and education implementation. However, despite the challenges of expanding care teams, the addition of GPOs in cancer care provision has been tremendously valuable in the Canadian setting, lending confidence to the development of a national GPO training program in Nepal that focuses on competencies relevant to the Nepalese cancer care system. The contributions GPOs bring to cancer care teams provide new mechanisms to manage an increasing global cancer burden by strengthening the workforce.

## Funding declaration

This work was supported by Conquer Cancer Foundation Young Investigator Award in Global Oncology.

## Conflicts of interest

Dr. Gyawali has received consulting fees from Vivio Health, unrelated to the manuscript. The other authors have no conflicts of interest to disclose.

## Figures and Tables

**Table 1.** Demographic characteristics of respondents.

		Canada *N* = 48	Nepal *N* = 7
Variable	Categories	Percentage	Percentage
Gender	Man	45.8	42.9
	Woman	54.2	57.1
	I do not identify within the gender binary	0.0	0.0
	I prefer not to disclose gender information	0.0	0.0
Age	<25	0.0	0.0
	25–34	4.2	0.0
	35–44	43.8	100.0
	45–54	18.8	0.0
	55–64	27.1	0.0
	≥65	6.3	0.0
Years in practice	<5	25.0	71.4
	6–10	18.8	14.3
	11–15	12.5	0.0
	16–20	10.4	14.3
	20–25	14.6	0.0
	26–30	10.4	0.0
	>30	8.3	0.0
Current practice setting	Large population centres (>100,000)	95.8	N/A
	Medium population centres (30,000–99,999)	4.2	N/A
	Small population centres (1,000–29,000)	0.0	N/A
	Rural area (<1,000)	0.0	N/A
Current practice setting	Metropolitan	N/A	100.0
	Sub-metropolitan	N/A	0.0
	Municipality	N/A	0.0
	Village Development Committee (VDC)	N/A	0.0
Training setting	Large population centres (>100,000)	100.0	N/A
	Medium population centres (30,000–99,999)	0.0	N/A
	Small population centres (1,000–29,000)	0.0	N/A
	Rural area (<1,000)	0.0	N/A
	Metropolitan	N/A	100.0
	Sub-metropolitan	N/A	0.0
	Municipality	N/A	0.0
	VDC	N/A	0.0
Are you affiliated with a medical college?	Yes	97.9	71.4
	No	2.1	28.6
Current position^a^	Medical officer	N/A	0.0
	Senior medical officer	N/A	0.0
	Consultant	N/A	16.7
	Senior consultant	N/A	0.0
	Lecturer	4.2	16.7
	Assistant professor	41.7	16.7
	Associate professor	35.4	33.3
	Professor	12.5	16.7
	Other	6.3	0.0
What is the status of your institution?	Public/Government	93.8	100.0
	Private	0.0	0.0
	Both public and private	0.0	0.0
	Medical college	6.3	0.0
	Other	0.0	0.0
My team is made up of the following care providers.			
(Select all that apply)	Nurses	97.9	100.0
	Nurse practitioners	85.4	50.0
	Occupational therapists	31.3	50.0
	Pharmacists	97.9	83.3
	Social workers	83.3	33.3
	Psychologists	39.6	50.0
	Medical oncologists	97.9	83.3
	Radiation oncologists	89.6	50.0
	Surgical oncologists	83.3	83.3
	Haematologists	60.4	66.7
	I do not work in a team	2.1	0.0
	Other:	6.3	16.7
	Clerical, Secretarial, GPO,		
	Palliative care/Symptom control		
There are regular multi-disciplinary case	Yes	100.0	100.0
Conferences/tumour boards at my institution	No	0.0	0.0

a Positions are not mutually exclusive, so the sum is greater than 100%

**Figure S1.**
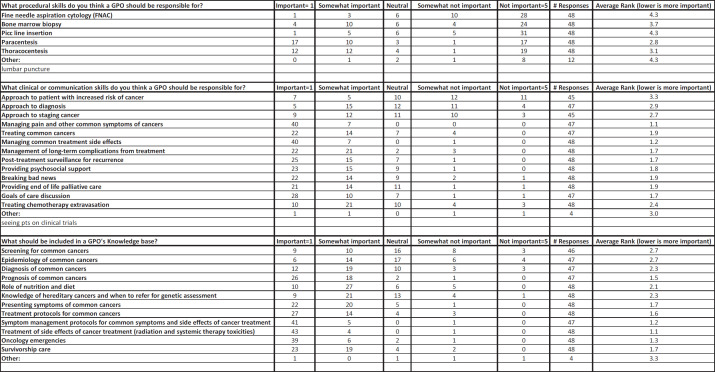
Canadian oncologist’s ranking of procedural, clinical/communication skills and GPO knowledge domains.

**Figure S2.**
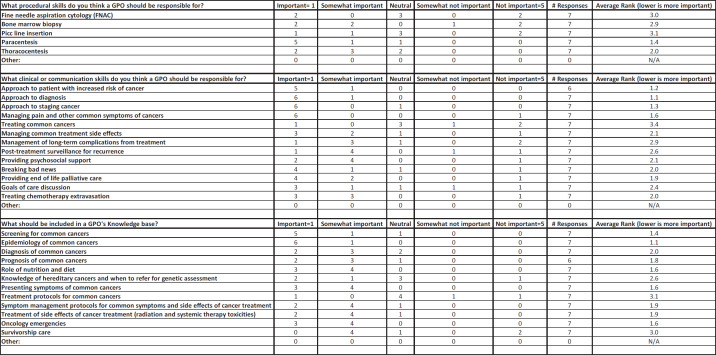
Nepalese oncologist’s ranking of procedural, clinical/communication skills and GPO knowledge domains.
